# Tools to compare antibody gold nanoparticle conjugates for a small molecule immunoassay

**DOI:** 10.1007/s00604-023-05637-x

**Published:** 2023-01-20

**Authors:** Monika Conrad, Günther Proll, Esteban Builes-Münden, Andreas Dietzel, Sven Wagner, Günter Gauglitz

**Affiliations:** 1 Institute of Physical and Theoretical Chemistry (IPTC), Eberhard Karls Universität Tübingen, Auf der Morgenstelle 18, 72076 Tübingen, Germany; 2 Institute of Microtechnology, Technische Universität Braunschweig, Alte Salzdahlumer Straße 203, 38124 Braunschweig, Germany; 3 OFFIS—Institut für Informatik, Escherweg 2, 26121 Oldenburg, Germany

**Keywords:** Antibody-conjugated nanoparticle, Binding inhibition assay, Functionalization, Gold nanoparticles, Lateral flow assay, Small molecules detection

## Abstract

Antibody gold nanoparticle conjugates as recognition elements are essential for the overall performance of lateral flow assays. When immobilizing antibodies on gold nanoparticles, the challenge is to prevent aggregation and to ensure that the antibodies are correctly oriented so that they remain functional and their paratopes remain accessible. There are many methods available, and it is difficult to decide which one to use. To help selecting the most appropriate conjugate production method, different synthetic routes of binding antibodies to gold nanoparticles are systematically investigated for the purpose of a quantitative lateral flow test for small molecules. The direct comparison of different conjugate syntheses shows how to select a suitable conjugate for a lateral flow assay. The syntheses examined are direct adsorption of antibody, direct adsorption of reduced antibody, covalent binding to polyethylene glycol linker, and binding via biotin-streptavidin interaction. The conjugates are characterized using UV-Vis spectroscopy and dynamic light scattering to determine their stability. Their performance on structured lateral flow test strips is examined using calibrations for different amitriptyline concentrations. It was shown that the best conjugate for quantification of amitriptyline was realized by direct adsorption of an UV-light irradiated antibody to gold nanoparticles. The methods employed can serve as a guide for selecting the most appropriate conjugate for an application and enhance the performance of lateral flow assays.

**Graphical abstract:**

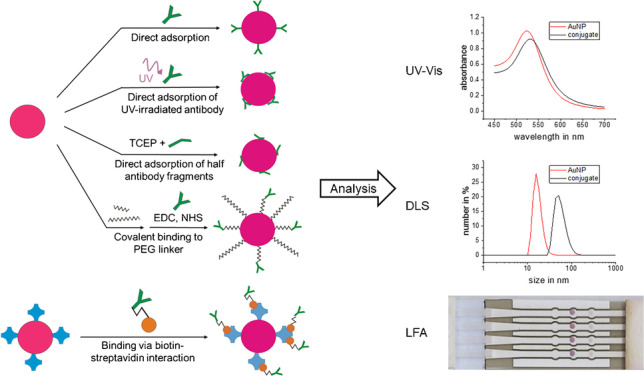

**Supplementary Information:**

The online version contains supplementary material available at 10.1007/s00604-023-05637-x.

## Introduction

Lateral flow assays (LFAs) are a great analytical tool for point-of-care-testing (POCT) [1, 2]. To use LFAs in therapeutic drug monitoring (TDM), they should provide quantitative results [3]. For POCT, these quantitative assays are not only needed for large analytes typically analysed in sandwich assays, but also for analytes of low molecular weight (<1000 Da).

POCT is especially important in the field of antidepressants as these show a narrow therapeutic window, and it should be regularly checked whether the patients receive the correct dose [4]. The analyte amitriptyline (AMT) was chosen as an example for an antidepressant where TDM is beneficial for patients [5]. The conventional sandwich assay is not applicable for developing an LFA for AMT because only one antibody can bind to a small molecule like AMT at a time, not two, as would be necessary for a sandwich assay. Instead, the binding inhibition test is used as it allows the quantification of small molecules. For the development of an LFA, choosing the recognition element is an important step [6, 7]. Functionalization of gold nanoparticles (AuNP) with antibodies is a common way to obtain recognition elements for LFAs [8, 9].

**Fig. 1:**
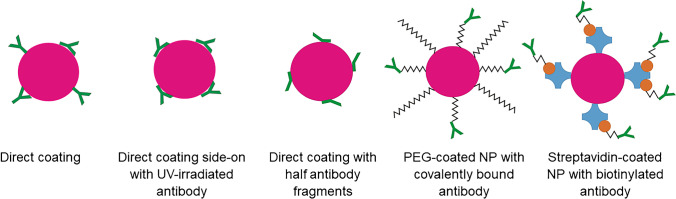
Schematic picture of different strategies for binding antibodies to AuNP: direct coating, where the unmodified antibody is randomly attached to the surface of the AuNP; direct coating with UV-activated antibody which leads to oriented binding; direct coating with half antibody fragments where the antibody is chemically reduced by TCEP; PEG-coated NP with covalently bound antibody where the AuNP is pegylated and the antibody subsequently attached via a peptide bond; Streptavidin-coated AuNP with biotinylated antibody, where biotin is bound to the antibody via a spacer

The aim of this work is to find the optimal strategy for immobilizing anti-AMT antibodies on gold nanoparticles using AMT as an example analyte. This process is accompanied by analytical methods and evaluations that are transferable to related problems and thus provide guidance for an optimal result in each individual case. This approach will help in finding the best antibody-gold nanoparticle conjugate for our model system which is a quantitative binding inhibition assay for AMT. There are many methods for binding antibodies to gold nanoparticles. The methods investigated here are direct coating in two buffers, coating with UV-irradiated antibody, coating with TCEP-reduced antibody, binding of antibody via PEG-linker, and binding via streptavidin-biotin interaction (Fig. 1).

Some of these conjugates have been compared before. Di Nardo et al. [10] synthesized conjugates by direct coating, covalent binding of antibody to a polymer, and binding of antibody via protein A on AuNP. They received comparable results for all three conjugates on their lateral flow immunoassay and observed that the sensitivity depends on the amount of antibody bound to AuNP. Mustafaoglu et al. [11] compared direct coating, covalent binding via PEG-linker, coating with reduced antibody, and the UV-NBS method. They obtained enhanced antigen detection efficiency with the UV-NBS method. A comparison of as many conjugate techniques as in this paper has so far not been carried out. For characterization of antibody AuNP conjugates, UV-Vis spectroscopy and dynamic light scattering (DLS) are methods typically employed [10–13], and as they are often available and easy to perform, they will be used to characterize the synthesized conjugates of this study.

When directly binding the unmodified antibody to AuNPs, the antibodies adsorb onto citrate-capped AuNPs via non-covalent interactions [14]. These include ionic interaction between negatively charged nanoparticle and positive charge on the antibody, and hydrophobic interaction between antibody and metal surface, followed by the formation of a dative bond between metal and sulphur atoms of the antibody [15]. Direct coating leads to randomly oriented antibodies on the gold surface. It was shown that pH 7.5 leads to more accessible antigen-binding sites than higher pH [12], while at lower pH electrostatic bridging leads to aggregation of AuNP [13]. Direct coating is often performed in phosphate buffer [12, 13], but since phosphate can act as complexing agent, usage of other buffer salts might improve antibody binding. The direct coating in PBS is compared to that in tris(hydroxymethyl)aminomethane (TRIS) buffer, both at pH 7.4.

To improve the direct adsorption of antibodies onto gold surfaces, the antibody can be irradiated by UV light resulting in disulphide bridges breaking in the environment of tryptophan [16]. The four disulphide bridges affected by photochemical reduction are located in the constant region of the light chain and in the constant region of the heavy chain [17]. The opened disulphide bridge leads to better binding to the gold surface and to more exposed Fab fragments favouring oriented binding [18]. Atomic force microscopy showed that after UV irradiation, side-on orientation of antibodies is favoured where one Fab domain is protruding [19] ensuring accessible antigen binding sites.

Furthermore, antibodies can be chemically reduced resulting in antibody fragments with exposed native thiol groups which chemisorb onto the gold surfaces [20]. MALDI-MS analysis showed that using the mild reducing agent tris(2-carboxyethyl)phosphine (TCEP) mainly reduces the disulphides in the Fc region and results in half antibody fragments keeping the paratopes intact [20]. In addition TCEP has the advantage that it contains no competing thiol groups; thus, no separation step is needed [21].

An alternative approach is coating the AuNPs with a linker molecule to which the antibody is bound via a covalent bond. As a linker molecule, bifunctional polyethylene glycol (PEG) is suitable because longer surface-bound ligands stabilize the gold colloid due to increased steric repulsion [22]. When using HS-PEG-COOH, the citric acid on the gold nanoparticle surface is exchanged by the stronger binding thiol group, and the carboxyl group is activated via EDC/NHS chemistry and reacts with primary amines of the antibody. Mixed PEG layers of high and low molecular-weight PEG combine good colloidal stability with a high level of bioconjugation [23]. Usually EDC/NHS chemistry is performed in pH 6 or 6.5 because of the stability of the activated intermediate, but it was found that lower pH of 5.5 favours the immobilization of antibody because of increased electrostatic repulsion at higher pH [24]. For oriented immobilization, the pH is reduced to 5 which leads to a high density of positive charges on the major plane of the antibody to which the negative COOH-PEG-AuNP intermediate binds [25].

Another method uses the streptavidin-biotin interaction. Streptavidin-coated nanoparticles can be used since streptavidin shows a high affinity for binding biotin (*K*_d_ = 10^−15^ M) [26] which is often used in bioanalytical applications [27]. Biotin can be easily conjugated to antibodies [28]. It is recommended to include a spacer between antibody and biotin to avoid steric hindrance because the four biotin binding pockets of streptavidin are buried inside [29].

In this paper, we will compare the methods mentioned above for binding antibodies to AuNPs. There are additional methods available like the use of protein A/G to bind antibodies to gold nanoparticles which can be also compared by the methods used in this paper. The scope of this paper is not to optimize each of the individual approaches of binding antibodies to gold nanoparticles. Instead optimized procedures from other publications were taken, and the different methods were compared. This approach shows an example strategy for finding the best conjugation technique for an LFA. The synthesized conjugates are assessed based on their yield and the amount of agglomeration to judge their stability. Their function and performance on an LFA are demonstrated via quantitative calibration. The used analytical methods for characterizing the antibody gold nanoparticle conjugates can be transferred to related problems and help in finding the right method for the desired application.

## Materials and methods

### Materials

Common chemicals were purchased from Sigma-Aldrich (Darmstadt, Germany). Buffers used were 10 mM phosphate-buffered saline (PBS) pH 7.4 and pH 6.8, 10 mM tris(hydroxymethyl)aminomethane (TRIS) pH 7.4, 10 mM 2-(N-morpholino)ethanesulfonic acid (MES) pH 5 and pH 6, and 10 mM bicarbonate buffer pH 8. Milli-Q water was used in the preparation of all solutions. Gold nanoparticles (AuNP, 20 nm, optical density OD 1) were purchased from BBI solutions (Cardiff, UK). α-Thio-ω-(propionic acid) octa(ethylene glycol) HS-PEG(8)-COOH (459 Da) was purchased from Iris Biotech (Marktredwitz, Germany) and SH-PEG-COOH (3000 Da) from Rapp Polymere (Tübingen, Germany). EZ-Link™ NHS-PEG4-Biotin, No-Weigh™ Format was purchased from Thermo Scientific (Rockford, USA). Monoclonal antibody anti-amitriptyline clone 202 (anti-AMT) supplied in PBS pH 7.4 was purchased from Aviva Systems (San Diego, USA). Nortriptyline-bovine serum albumin (NRT-BSA) was purchased from CalBioreagents (San Mateo, USA). Polyclonal antibody goat anti-mouse IgG (multispecies adsorbed) was purchased from Bio-Rad Laboratories (Feldkirchen, Germany). Amitripyline-Ab-Gold (DCM-C, 40 nm) and streptavidin-gold (SA-AuNP, 40 nm) were provided by Microcoat Biotechnologie (Bernried, Germany).

### Gold nanoparticle conjugates

The binding kinetics of the employed antibody were previously characterized by reflectometric interference spectroscopy and showed suitable characteristics for being employed in an LFA with an association rate constant of 2.4 ∙ 10^4^ M^−1^ s^−1^ and dissociation rate constant of 1.3 ∙ 10^−3^ s^−1^ [30]. All syntheses of antibody gold nanoparticle conjugates were performed in Eppendorf Protein LoBind Tubes.

#### Direct coating in PBS/TRIS buffer (DCP-C/DCT-C, -C for conjugate)

The procedure for DCP-C and DCT-C is the same, except for the buffer solution used (PBS pH 7.4 for DCP-C and for TRIS pH 7.4 for DCT-C). Initially, a flocculation test according to Paek et al. [31] was conducted, and the UV-Vis spectra were recorded in the presence of salt to determine the optimal antibody concentration. One-millilitre 1 OD AuNP solution (20 nm) was mixed with 5 μl 1.2 mg ml^−1^ anti-AMT and incubated for 40 min at 300 rpm at RT. One hundred-microlitre 10 mg ml^−1^ BSA in buffer was added and incubated for 20 min. To remove excess antibody, three washing steps followed. The solution was centrifuged at 8000 g and 4 °C for 30 min. The supernatant was discarded, and the residue resuspended in 1 ml 1 mg ml^−1^ BSA by sonication. After the last washing step, the residue was resuspended in 0.1 mg ml^−1^ BSA to achieve a final volume of 1 ml. Finally, 100 mg sucrose was added. BSA stabilizes the conjugate, and sucrose ensures that the native conformation of the dehydrated proteins is preserved and a quick re-solubilization upon wetting.

#### Direct coating with UV-activated antibody (UV-C)

Five-microlitre 1.2 mg ml^−1^ anti-AMT was dissolved in 1 ml milli-Q water and irradiated (254 nm, E = 90 ∙ 100 μJ cm^−2^) using UVP Translinker CL-1000 UV in a small petri dish. One-millilitre 1 OD AuNP was added, and the following steps were the same as for DCT-C.

#### Direct coating with TCEP reduced antibody (TCEP-C)

The synthesis for direct coating with reduced antibody was based on [20]; 4.2 μl 1.2 mg ml^−1^ anti-AMT was reduced with 5 μl 5 mM TCEP in 40 μl PBS pH 6.8. This solution was incubated at RT at 300 rpm for 1 h. Then, 1 ml 1 OD AuNP (20 nm) was added, and the mixture was incubated at RT at 300 rpm for 2 h. To remove excess antibody, three washing steps followed as above with resuspension in 15 mg ml^−1^ BSA in PBS pH 6.8. After the last washing step, the residue was resuspended in 0.1 mg ml^−1^ BSA in PBS pH 7.4. Finally, 100 mg sucrose was added.

#### PEG coated nanoparticle with peptide bond via EDC-NHS-Chemistry (PEG-C)

The synthesis for PEG-C was based on [25]; 2.4 μl 1 mg ml^−1^ SH-PEG-COOH (459 Da) and 15.6 μl 1 mg ml^−1^ SH-PEG-COOH (3000 Da) in water were added to 1 ml AuNP (20 nm). The solution was incubated at 650 rpm and RT overnight. Three washing steps followed with centrifugation at 13,000 g for 20 min and resuspension in water. After the last washing step, the sediment was resuspended in 2 μl 10 mg ml^−1^ EDC and 3.6 μl 10 mg ml^−1^ sulfo-NHS in MES pH 5. After incubation at 650 rpm at 37 °C for 10 min, 500 μl MES pH 5 was added. Subsequently, the mixture was centrifuged at 13,000 g for 10 min, the supernatant discarded, and the residue resuspended in 16 μl 1.2 mg ml^−1^ anti-AMT and 400 μl MES pH 5. After incubation at 300 rpm at RT overnight, the mixture was centrifuged at 8000 g and 4 °C for 30 min, resuspended in bicarbonate buffer, and incubated at 300 rpm at RT for 30 min. After centrifugation (8000 g, 4 °C, 30 min), the residue was resuspended in 20 mg ml^−1^ BSA in MES pH 6. After incubation at 300 rpm at RT for 1 h, the mixture was centrifuged, the supernatant discarded, and the residue resuspended in PBS pH 7.4. Finally, 100 mg sucrose was added.

#### Streptavidin-coated nanoparticle with biotinylated antibody (SA-C)

Five-microlitre 1.2 mg ml^−1^ anti-AMT was dissolved in 50 μl PBS pH 7.4, and 0.47 μl 1 mg ml^−1^ NHS-PEO_4_-biotin was added. The solution was incubated for 1 h at RT. Excess biotinylation reagents were removed by washing three times with 400 μl PBS in centrifuge filters (AmiconUltra 0.5 ml Ultracel 30 K) spinning down at 8000 g for 20 min. The biotinylated antibodies were added to 1 ml streptavidin-gold (40 nm) diluted to 1 OD and incubated at RT and 300 rpm for 1 h. To remove excess antibody, three washing steps followed as for DCT-C conjugate.

### UV-Vis measurements

UV-Vis measurements were carried out via a Lambda 9 spectrometer using a standard 1-cm pathlength quartz cuvette. The reference was a cuvette filled with PBS. Spectra were obtained from 450 to 700 nm, with a spectral resolution of 1 nm.

### Dynamic light scattering

DLS is a common technique for determining the particle size in colloidal suspensions. The DLS measurements were carried out using a Zetasizer Nano ZS from Malvern Panalytical. The refractive index (RI) value used for the colloid particles was 0.135. The viscosity of the sample was assumed to be the viscosity of the dispersant water 0.8872 with an RI of 1.330. Measurements were carried out at 25 °C with an equilibration time of 10 s using a sample volume of 50 μl in disposable cuvettes (ZEN0040). Each sample was measured in triplicate with automatic measurement duration at a measurement angle of 173° (Backscatter), automatic positioning, and automatic attenuation selection. The wavelength of the HeNe laser was 633 nm. An average result of the triplicate measurements was created, and the number mean of the main peak taken as particle diameter. The DLS number distribution shows a good approximation of the size parameters, while the DLS intensity distribution is highly affected by the presence of agglomerates.

### Test strip preparation

**Fig. 2:**
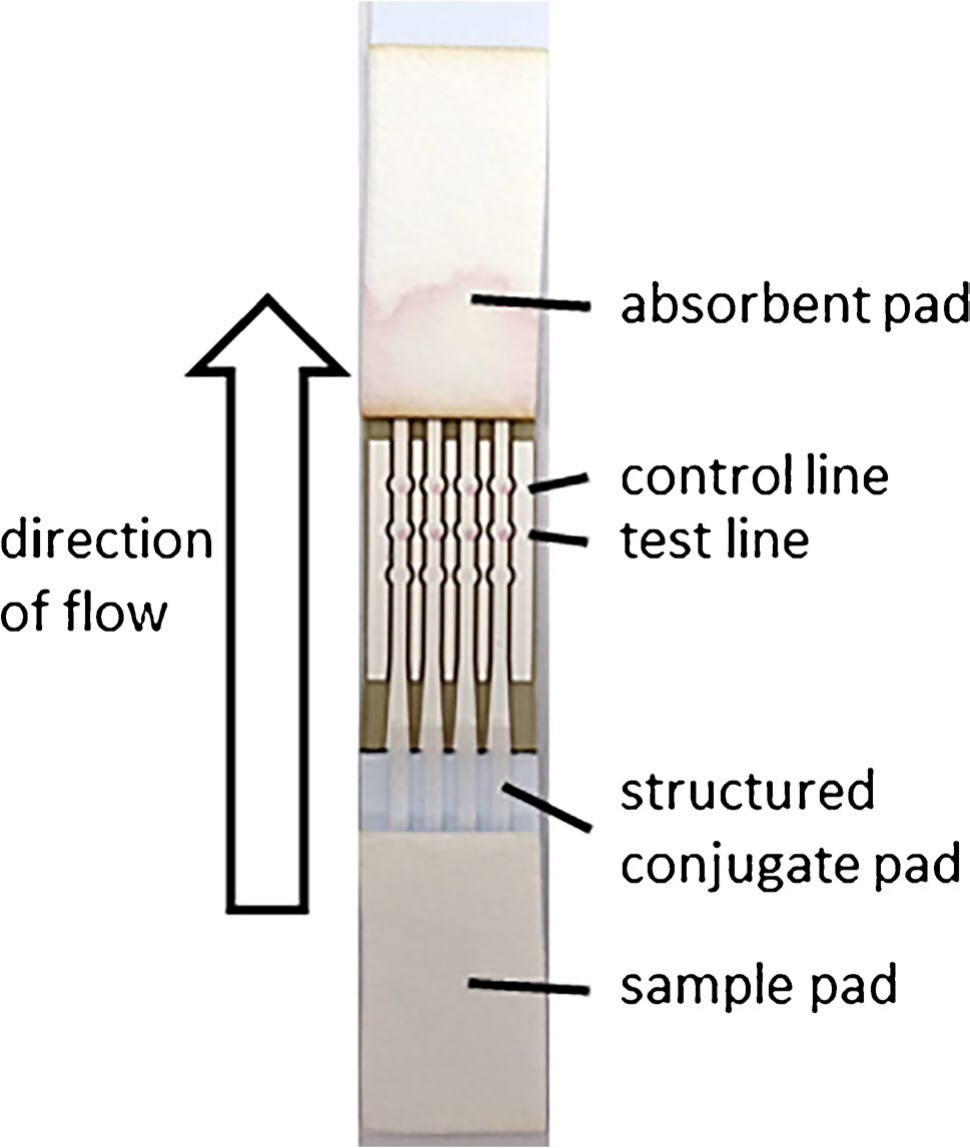
Structure of the lateral flow strips used. The sample is applied to the sample pad, which flows through the conjugate pad where the antibody gold nanoparticle conjugates were dried and suspends them. The solution migrates through the structured nitrocellulose membrane towards the test line where the antigen is immobilized. The excess probe reaches the control line, where the remaining antibody gold nanoparticle conjugates are captured by the secondary antibody

Each test strip consisted of sample pad, conjugate pad, nitrocellulose membrane, and absorbent pad (Fig. 2). These components were fixed on a PVC-backing card. To achieve multiple measurements on one strip, the strips featured four channels with three test zones. All channels share the same sample pad, structured conjugate pad, and absorbent pad. On the conjugate pad, the gold labelled detection antibodies are stored.

### Structuring of membranes

Nitrocellulose membranes (CN-140 UniSart, Sartorius, Göttingen, Germany) were structured using the cold laser ablation technique [32]. A frequency-doubled Yb:KGW-solid-state laser (Light Conversion Pharos. Vilnius, Lithuania) was focused on the nitrocellulose membrane emitting at 1030 nm wavelength with a repetition rate of 600 kHz, a laser power of 6.74 W, and a laser writing speed of 2400 mm s^−1^. A very high photon density was concentrated in a 34 μm laser spot hitting the material in ultra-short laser pulses of 212 fs. Because of the short pulse duration, the irradiated volume material changes directly from solid into the gas phase, without reaching the low material flash point. In this way, channel barriers were structured at a distance of 1000 μm (channel width), creating four channels that are used to increase the measurement reproducibility. Convexities at the test and control zone were also fabricated as described.

### Immobilization of proteins

For spotting anti-mouse on the control line (upper test zone) and NRT-BSA on the test line (middle test zone), a non-contact spotter (instrumentTWO, M2-Automation MDC07, Berlin, Germany) provided by Lilian Labs (Braunschweig, Germany) was used. Each spot consisted of two droplets of 40–50 nl 1 mg ml^−1^ protein solution. After the spotting process, the proteins were immobilized at 50 °C for 10 min.

### Pad preparation

The sample pads (SureWick CFSP, cellulose, Merck Millipore, Darmstadt, Germany) were impregnated with a solution containing 0.5% BSA and 0.25% Tween^®^20 in TRIS-buffer at pH 8.0 by shaking for 4 h. The pads were dried at RT overnight. The conjugate pads (SureWick GFCP, glass fibre, Merck Millipore, Darmstadt, Germany) were laser structured and impregnated in 10 mg ml^−1^ BSA in PBS pH 7.4 for 1 h and dried at RT overnight. Each of the four tips of the dried conjugate pads was treated with 4 μl OD 1 conjugate solution and dried at 37 °C for 1 h.

### Calibration

All components were assembled as shown in Fig. 2, using the adhesive strips on the backing card. Each segment overlapped its neighbouring segment to ensure migration of the solution during the assay. Calibration measurements were performed for AMT concentrations of 0, 1, 10, 30, 100, 300, 1000, and 10,000 μg l^−1^ in PBS; 250 μl of sample solution was applied on the sample pad. After the test strip was dried at RT, pictures were taken using a compact camera (RX100III, Sony, Tokyo, Japan) under artificial lighting (portable photo studio with LED strips 3400 lm, Samtian, Shenzen, China). The camera setting was shutter speed 1/100 s, aperture f3.5, ISO 100.

### Signal evaluation

A self-developed software was used to automatically evaluate the biosensor colour spots. The Deep Learning-based software makes use of the laser ablation structures on the biosensor surface to find the colour spots in the image. Specifically, we use a convolutional neural network as object detector to locate all test zones. The following steps to calculate the measurement signal of a test zone are based exclusively on the green colour channel of the image. Multiple reference points on the biosensor surface were used to account for local and global illumination variations. To decide which pixels belong to the red colour spot, a segmentation based on the Otsu thresholding method [33] was implemented. The final signal is the difference between the mean intensity of the red colour spot and a reference zone directly in front of it (between sample pad and spot). The signal of the test line was divided by the signal of the control line. Due to four channels on one strip, multiple measurements were performed on one strip enabling statistical analyses. The average signal of the four channels was calculated, and the signals of one calibration measurement were normalized using the signal of 0 μg l^−1^ AMT. A logistic function was fitted to the values with Origin 2021.

$$y={A}_2+\frac{A_1-{A}_2}{1+{\left(\frac{x}{x_0}\right)}^p}$$

The minimum detectable concentration (MDC) was calculated as the intersection of the lower limit of the 95% confidence band at the zero concentration with the calibration curve and the reliable detection limit (RDL) as the intersection point with the upper limit of the confidence band [34].

## Results and discussion

From the ratio of chemicals, it follows, that 34 antibodies were used per gold nanoparticle for DCP-C, DCT-C, and UV-C, 29 for TCEP-C, which is a two- to threefold excess compared to the number of antibodies that could theoretically fit onto the surface of the nanoparticle. Thus, it is assumed, that the surface of the nanoparticle is completely covered by antibodies. For PEG-C the ratio was 110 antibodies per nanoparticle which is an even greater excess. For SA-C six biotinylated antibodies per streptavidin coated nanoparticle are used in the reaction which means that unsaturated streptavidin binding sites are present, but this should not affect the functionality of the conjugate.

### UV-Vis spectroscopy

**Fig. 3:**
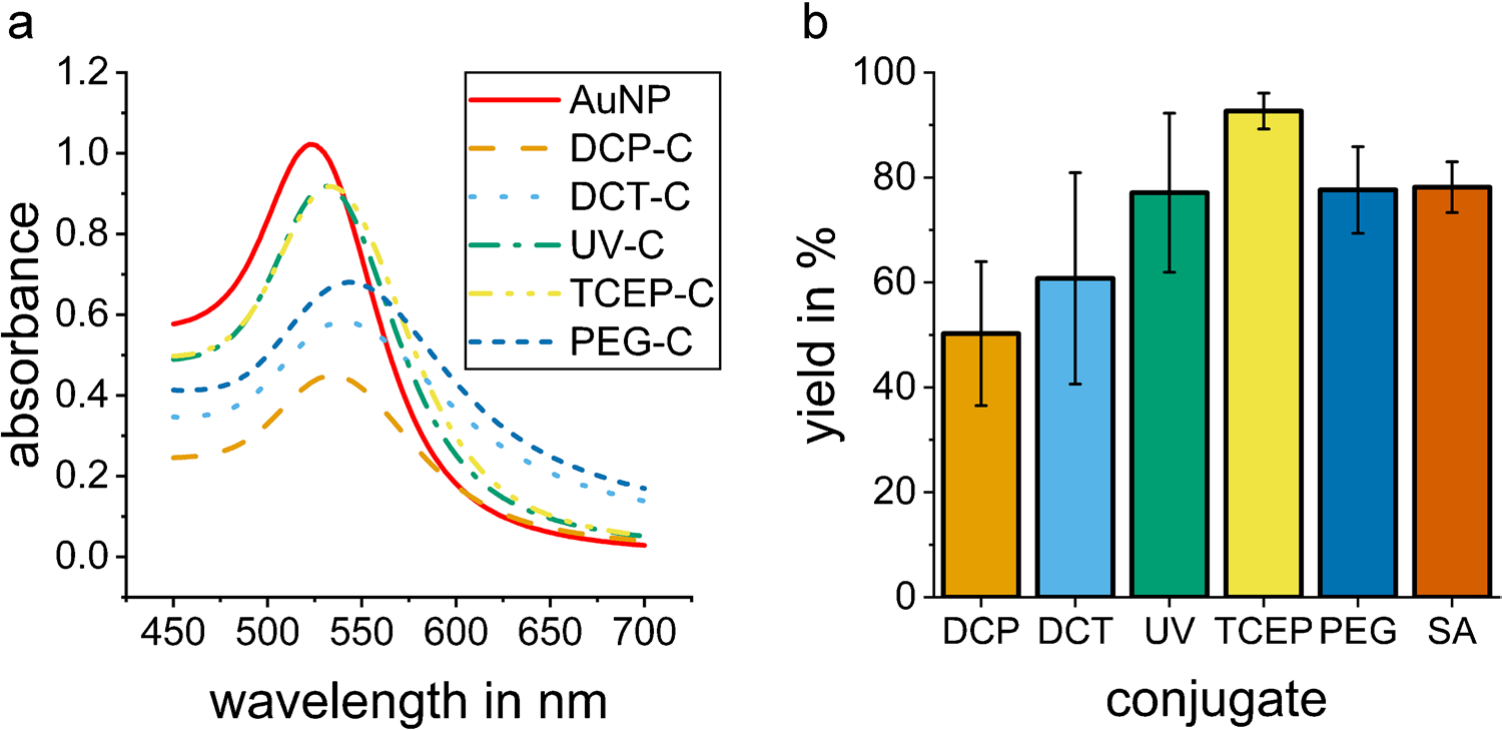
**a**) Examples of UV-Vis spectra of AuNPs (red line) and different conjugates, **b**) yield for conjugate syntheses calculated from the maxima of the UV-Vis absorbance spectra. The columns show the mean value of three syntheses with the standard deviation as error bars (*n*=3)

To characterize the synthesized conjugates, UV-Vis measurements were carried out. The UV-Vis measurements of AuNPs show a strong absorption at 522 nm. If the nanoparticles are covered with antibody, the maximum absorption wavelength is shifted to higher wavelengths [35]. The thicker the adsorbed layer, the larger the shift to higher wavelengths. The UV-Vis spectra of the different conjugates (Fig. 3a) show that all employed methods lead to an increase of the maximum wavelength. For DCP-C and PEG-C, the shift of the wavelength of maximum absorbance is less reproducible than for the other conjugates (Fig. S1). This shift is smaller for the conjugates UV-C and TCEP-C where half antibody fragments are bound to the AuNPs. For the conjugates DCP-C, DCT-C, and PEG-C, a peak widening is observed in addition to a shift of the absorbance maximum to higher wavelengths.

The increase of the wavelength of maximum absorption indicates that the antibody was successfully attached to the nanoparticle. The UV-Vis spectra measured after each individual step in the conjugate synthesis show that the wavelength shift occurs after addition of the antibody (Fig. S2.). Agglomeration that occurs during the synthesis leads to a larger shift of the absorption wavelength and to wider peaks. Thus, more agglomeration occurs in case of the syntheses DCP-C, DCT-C, and PEG-C. The shift of the wavelength of maximum absorbance is related to the thickness of the coating layer [36]. As the adsorbed layer is thinner for half antibody fragments, the conjugates UV-C and TCEP-C show the smallest shift of maximum wavelength. The shift of the maximum length and the peak broadening in the spectrum of PEG-C is not caused by swelling of the PEG because after formation of the PEG layer the UV-Vis spectra is similar to that of AuNP. Thus, aggregation occurs during the binding step with the antibody.

Besides, the maxima of the UV-Vis spectra show the yield that is achieved. All syntheses were started with 1 ml AuNP at OD 1. The optical density achieved at the end, when brought back to a volume of 1 ml, indicates how many nanoparticles were lost. More losses occur for conjugate syntheses DCP-C, DCT-C, and PEG-C (Fig. 3b).

Loss of AuNPs is probably caused by nanoparticles adsorbing to the wall of the reaction cup, by agglomeration due to centrifugation, or by the washing steps. The conjugates UV-C, TCEP-C, and SA-C were synthesized with the highest yield and show the most reproducible spectra, indicating that coating the AuNP with UV-irradiated antibody, half antibody fragments, or streptavidin offers greater stability. All in all, the replicate syntheses show the most reproducible results for TCEP-C regarding the shift of the wavelength and yield. This conjugate also achieves the highest yield.

### Dynamic light scattering

**Fig. 4:**
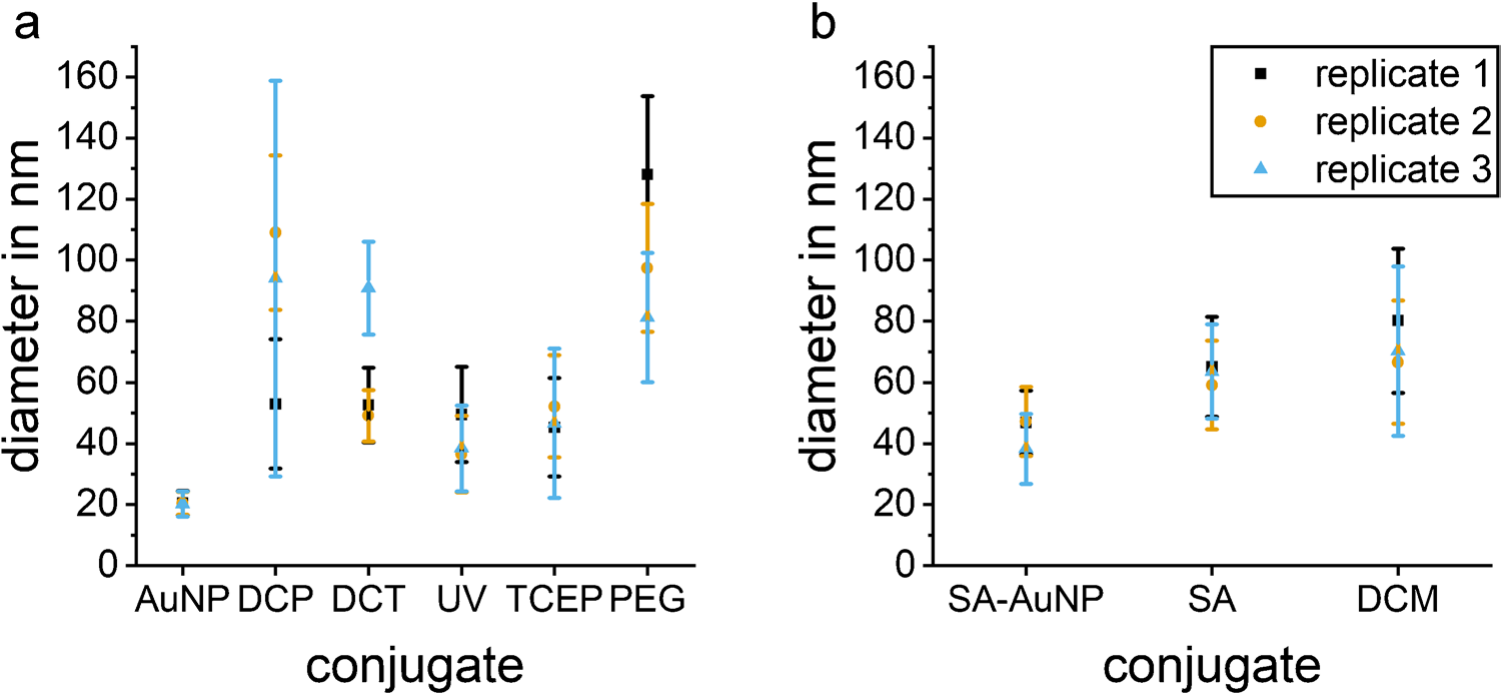
Hydrodynamic diameter from triplicate DLS measurements of the different conjugates: **a** starting material AuNP (20 nm), direct coating in PBS (DCP-C), direct coating in TRIS (DCT-C), direct coating with UV-activated antibody (UV-C), direct coating with TCEP-reduced antibody (TCEP-C), covalently bound antibody to PEG layer on AuNP (PEG-C); **b** starting material streptavidin gold (SA-AuNP, 40 nm) for conjugate with biotinylated antibody (SA-C), and direct coating of 40 nm AuNP by Microcoat (DCM-C). The averaged results of three DLS measurements of each conjugate are given with the error bar showing the standard deviation (*n*=3)

The size of the conjugates was determined by DLS to check whether the particles agglomerate. The DLS measurements show a hydrodynamic diameter of 22 nm for the employed AuNPs. An increase of the hydrodynamic diameter after coating the AuNPs with antibodies is observed for all syntheses (Fig. 4). The distribution of sizes shows that for DCP-C and PEG-C a broader distribution is obtained, while the size distributions for UV-C, TCEP-C, SA-C, and DCM-C show similar width. The polydispersity index shows a broad size distribution (PdI > 0.4) for DCT-C and UV-C, while the other conjugates display moderate polydispersity (Table S1). The conjugate UV-C has the smallest mean hydrodynamic diameter with 42 nm which is an increase of the hydrodynamic diameter of approximately 20 nm and a layer thickness of 10 nm.

The DLS measurements show an increase of the particle diameter for all syntheses, indicating that a layer has formed on the surface of the AuNPs, and the synthesis thus was successful. Assuming an antibody monolayer thickness of 15 nm, it can be concluded that a monolayer of antibody for DCP-C and DCT-C was formed which would correspond to ten antibodies per nanoparticle (calculated with an antibody surface area of (14.5 ∙ 8.5) nm^2^ [37]. This means that antibodies were added to the reaction in triple surplus. For UV-C and DCT-C, the thickness is less, but this can be explained by different orientation of the antibody or that the antibody was fragmented by the reduction. For PEG-C, the thickness is higher because PEG and antibody contribute to the layer. For SA-C, only few antibodies have been attached to the surface, six antibodies per nanoparticle were used while the surface area could accommodate 40. Thus, more antibodies could be attached to SA-AuNP.

When comparing the size distribution for the direct coating of the unmodified antibody in two different buffers (DCP-C in PBS and DCT-C in TRIS), DCP-C shows stronger agglomeration of the particles. This can be due to insufficient stabilization of the particles in PBS, indicating that TRIS improves stability of the conjugates. The increase in diameter of PEG-C (synthesized by forming a PEG layer first and afterwards covalently binding the antibody to it) is significantly larger than for other conjugates, and the size distribution of PEG-C is broad. The presence of bigger particles contributes to an increased light scattering, shifting the measured particle size towards larger values. As covalent bonds are formed in PEG-C synthesis, the AuNPs might become linked by several PEGs of different AuNP binding to several amino groups of the same antibody, leading to agglomerates. The particle size distribution shows good results with minor agglomeration for conjugates UV-C, TCEP-C, SA-C, and DCM-C.

### Conjugates on LFA

The different conjugates were tested on LFAs. Since the analyte is a small analyte, a binding inhibition assay is used, where the analyte is incubated with the detection antibody bound to the gold nanoparticle. Only the remaining free detection antibodies which are not inhibited by the analyte can bind to the antigen immobilized on the test line. Comparison of the signal of the conjugate without inhibition where buffer without AMT was applied as sample is shown in Fig. S3. The tests of the conjugates on the test strips showed that the signal intensities obtained for conjugates PEG-C and SA-C were less than for the other conjugates. For example, the zero signal for UV-C is three times larger than for PEG-C and twice as high as for SA-C. For DCT-C and UV-C, one batch showed much higher signals than the other batches, while for the other conjugates different batches give similar signal intensities.

The very high signal intensities in one batch of UV-C and DCT-C could be the result of spotting because the membranes were not spotted in one batch. Possibly the drops formed during spotting were of slightly different sizes leading to more antigen on the test line and consequently to more accessible binding sites for the conjugate. The conjugates PEG-C and SA-C show reduced signal intensity. As these two conjugates were prepared by chemical modification of the antibody, a reason for the reduced signal intensity might be low yield of the modification or that the formation of the peptide resulted in functionality loss of the antibody. In case of SA-C, six antibodies per gold nanoparticle were used in the reaction, but it is possible that not all antibodies were biotinylated or that antibodies were lost in the filtration process before attaching them to streptavidin-gold resulting in low signal. In case of PEG-C, an excess of 110 antibodies per nanoparticles was used; thus, it is unlikely that there were not enough antibodies which could bind to the pegylated nanoparticle, but it is possible that the reagents for the peptide bond formation were not completely removed by the washing step which might cause peptide bond formation between antibodies and thus agglomeration.

**Fig. 5:**
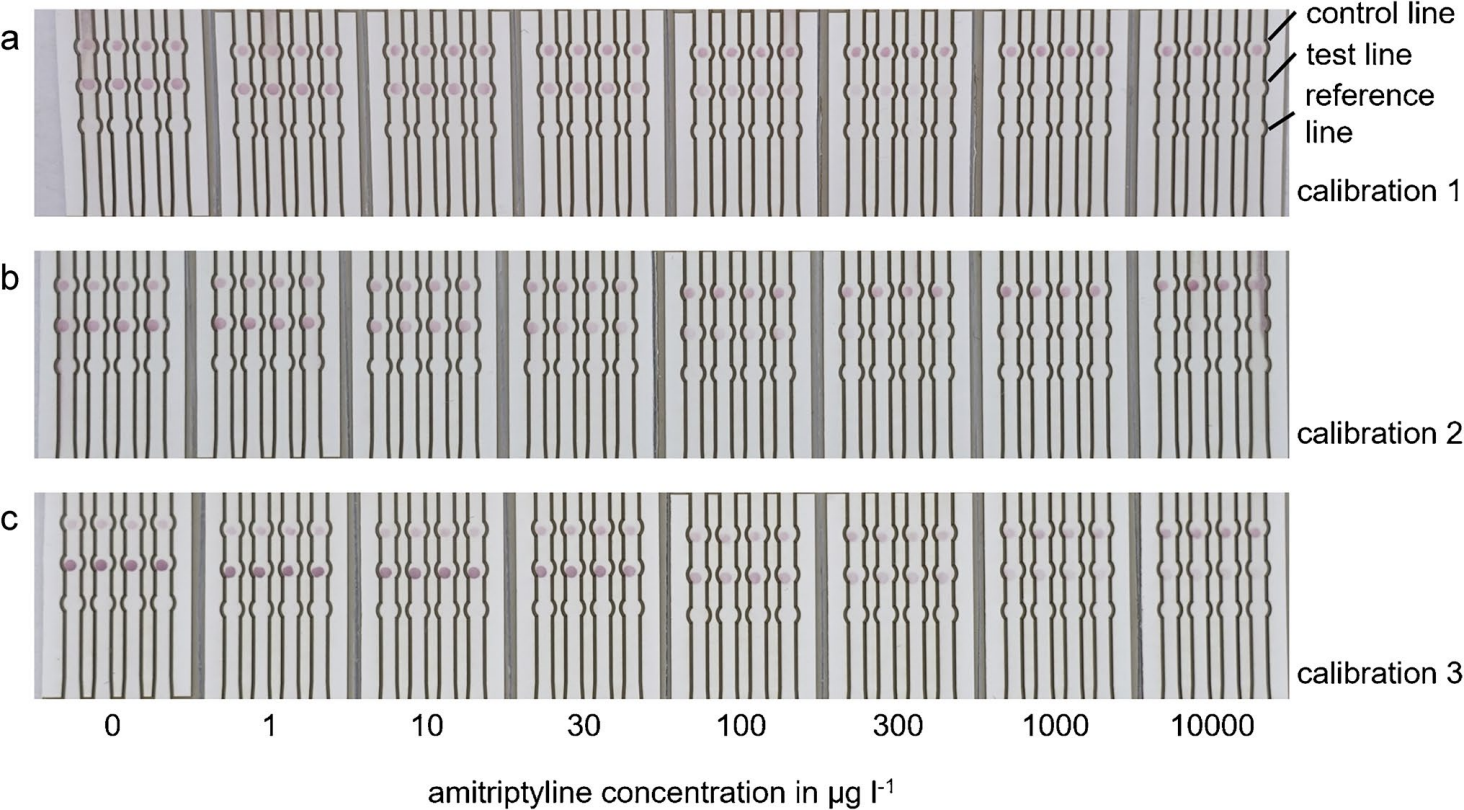
Test strips of triplicate calibration using UV-C conjugate with different AMT concentrations in the sample (from left to right) 0, 1, 10, 30, 100, 300, 1000, 10,000 μg l^−1^. Pictures were taken with a compact camera under artificial lighting

**Fig. 6:**
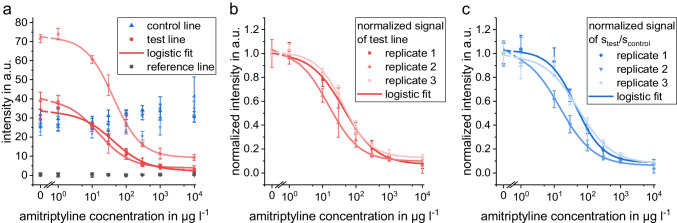
Triplicate calibration curves using UV-C with logistic calibration curve for eight concentrations, **a**) signal intensities of control line, test line, and reference line as mean with standard deviations as error bars of the four channels on each strip (*n*=4), **b**) normalized signal of test line (s/s_0_), **c**) normalized signal of test/control. The calibration curves were calculated using a four-parameter logistic fit function

An example for triplicate calibration is shown in Fig. 5. It is obvious that intensities in test and control line on one strip show very good intra chip reproducibility. When comparing triplicate calibrations, it is noticeable that the intensities of test and control line show different intensities for different batches. To allow comparison of different calibrations, the signals of the test line were normalized using the signal of the zero concentration (Fig. 6). In the case of UV-C conjugate, normalization leads to similar signals and curves for the triplicates. As the signal in the control line increases with higher concentrations, the signal of the test line was divided by the signal of the control line and subsequently normalized utilizing the zero concentration. These signals show good agreement between batches; thus, a good inter-chip reproducibility can be achieved by normalization. These signals were used to calculate calibration curves with a logistic function. The calibration curves for the other conjugates are shown in Fig. S4.

**Fig. 7:**
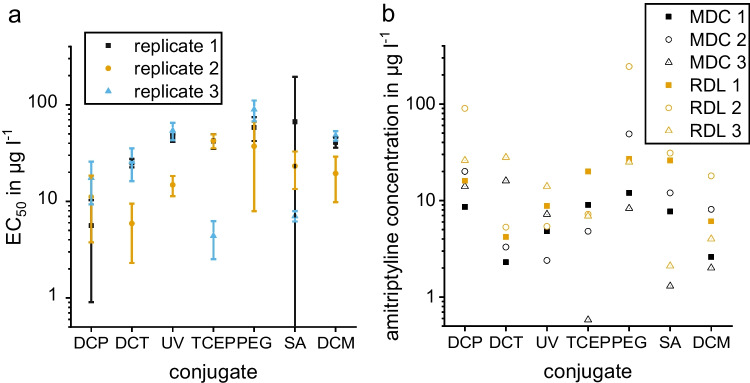
**a**) EC_50_ for triplicate calibrations of different conjugates with standard error as error bar, **b**) MDC (black) and RDL (yellow) for triplicate calibrations of different conjugates with evaluation of normalized signal of test/control. The corresponding calibration curves are shown in Fig. S4

**Fig. 8:**
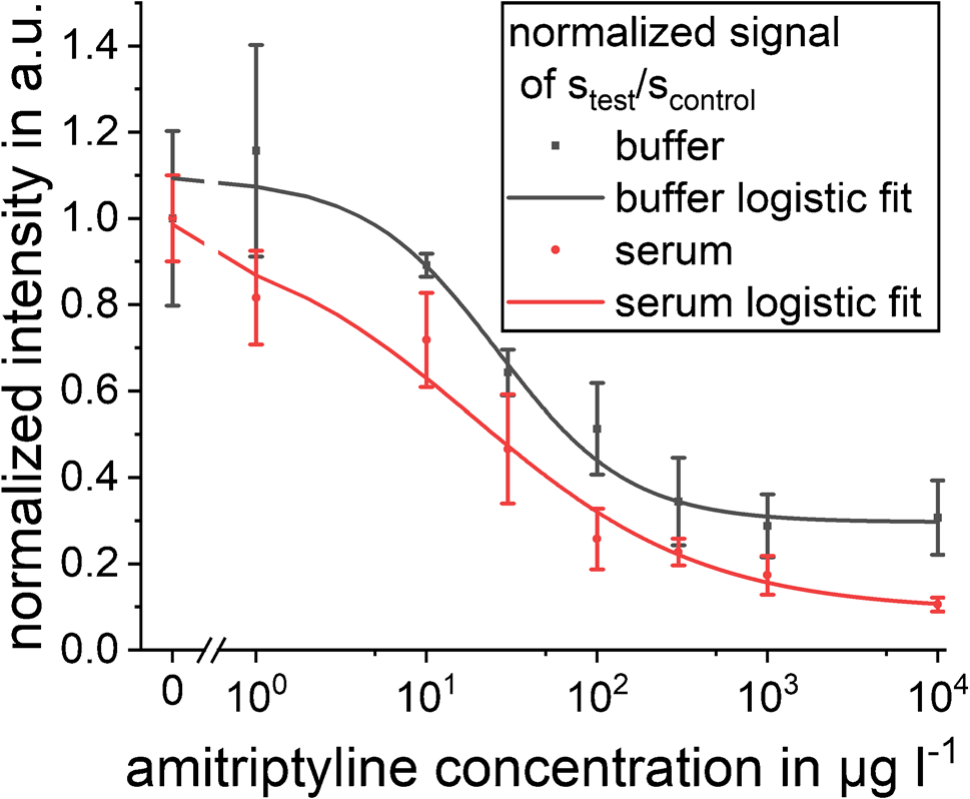
Calibration with amitriptyline in buffer (black) and in 1:10 serum in buffer (red) for eight amitriptyline concentrations. The signal of the test line was divided by the signal of the control line and normalized to the blank signal. The mean and standard deviation shown as error bars were calculated from the signals in six parallel channels (*n*=6)

The obtained half maximal effective concentration (EC_50_) (Fig. 7a), MDC, and RDL show that the smallest variation between batches is obtained for UV-C and DCM-C. The EC_50_ for conjugate SA-C has a very large error bar because of weak signals and lacking dynamics. The same applies to a lesser extent to DCP-C and PEG-C. These conjugates also show high variation between batches when calculating the MDC and RDL for the triplicate calibrations. UV-C and DCM-C show similar MDC and RDL for all three batches. Thus, UV-C and DCM-C show the best results, and their MDC is 5 μg l^−1^ which is suitable for the application of quantifying AMT in serum samples as the AMT concentration should be 100–200 μg l^−1^ [5]; thus, serum samples could be diluted tenfold before quantification. To investigate, if our conjugate behaves similar in serum and buffer samples, we recorded a calibration in serum where we used our further developed test strip with six instead of four channels. The change of the sample solution from buffer to 1:10 fetal bovine serum in buffer showed that similar calibration curves for both sample solutions are obtained and that the change in matrix and viscosity does not interfere with the assay (Fig. 8).

For the best two conjugates UV-C and DCM-C, the long-term stability of the conjugates was investigated. As the conjugates are more stable in the dried state on the conjugate pad, the prepared conjugate pads were stored for more than 9 months at room temperature. The comparison of the test line intensity on a test strip where buffer was used as the sample demonstrates that for UV-C (56 ± 5) % intensity was obtained and for DCM-C (43 ± 5) % shown in Fig. S5. These results show that the conjugates are stable for a long time after immobilization on the conjugate pad and that they could be used in a practical application.

## Conclusion

The comparison of the conjugate techniques showed that antibodies can successfully bind to AuNPs via different routes. Losses during syntheses can have several causes, and a lower yield was obtained for DCP-C than for other conjugates. DCP-C and PEG-C also showed a higher aggregation propensity which can be a reason for small yield. Aggregation can occur in case of insufficient stabilization of AuNP or because of antibodies linking several AuNP. DCP-C, PEG-C, and SA-C showed weak signals on test strips, indicating that the antibody was not correctly oriented for binding and antigen binding sites were inaccessible. Overall, the conjugates UV-C and DCM-C showed very good results. We recommend activation of the antibody by UV-light before conjugation as this improves the conjugate stability, gives higher yield, and is reproducible. The immunoassay for AMT with conjugate UV-C shows an MDC of 5 μg l^−1^, while a limit of detection of 540 ng l^−1^ [38] was achieved using reflectometric interference spectroscopy. Since patients experience an amelioration at plasma concentrations from 100 to 200 μg l^−1^ [5], it might be possible to dilute serum samples tenfold and subsequently analyse them using our immunoassay. Furthermore, the structured channels not only allow replicate measurements, but can also be used for multiparametric immunoassays [39] which allows further applications.

The strategy used to find the best conjugate for our application can serve as a guideline for finding the best way of binding an antibody to AuNP for any LFA. UV-Vis spectroscopy is performed to determine the yield of conjugate synthesis and can indicate whether binding was successful or agglomeration occurred. The size of the particles can be confirmed by DLS measurements. The final test on LFA close to the application shows the suitability of the conjugate for the given analytical problem.

## Supplementary information

ESM 1 (DOCX 2184 kb)

## Data Availability

The datasets generated during and/or analysed during the current study are available from the corresponding author on reasonable request.
