# Supplementary material for: Tools to compare antibody gold nanoparticle conjugates for a small molecule immunoassay
Source: Mikrochim Acta. 2023 Jan 20;190(2):62. doi: 10.1007/s00604-023-05637-x (PMC9859872; doi:10.1007/s00604-023-05637-x)
Supplement: Supplementary file 1 — (DOCX 2184 kb) [file 604_2023_5637_MOESM1_ESM.docx]

Supplementary Information (SI)

Tools to Compare Antibody Gold Nanoparticle Conjugates for a Small Molecule Immunoassay

Microchimica Acta

Monika Conrad^a^*, Günther Proll^a^, Esteban Builes-Münden^b^, Andreas Dietzel^b^, Sven Wagner^c^, Günter Gauglitz^a^

monika.conrad@uni-tuebingen.de

^a^Institute of Physical and Theoretical Chemistry (IPTC), Eberhard Karls Universität Tübingen, Auf der Morgenstelle 18, 72076 Tübingen, Germany

^b^Institute of Microtechnology, Technische Universität Braunschweig, Alte Salzdahlumer Straße 203, 38124 Braunschweig, Germany

^c^OFFIS - Institut für Informatik, Escherweg 2, 26121 Oldenburg, Germany

**Fig. S1**: UV-Vis spectra for the conjugates synthesized in triplicate and their starting material

**Fig. S2**: UV-Vis spectra of DCT measured after each individual conjugate preparation step. Starting material AuNP (red line), after addition of anti-AMT antibodies to AuNP (AuNP + Ab, black dashed line), after addition of BSA in TRIS buffer (AuNP-Ab + BSA, orange dotted line), and after the three washing steps. The shift of maximum wavelength occurs after addition of the antibody, further steps do not affect the position of the maximum. After addition of BSA a peak at 280 nm appears. The inset shows the enlarged maxima of the UV-Vis spectra and the position of the maxima of AuNP (red line) and after addition of antibody (black dashed line).

**Table S1**: Conjugate characteristics. The maximum of LSPR band was calculated as the absorbance peak difference between conjugate and AuNP due to the addition of a new layer. The layer thickness was calculated as the (diameter of resulting conjugate from DLS-diameter of AuNP)/2. Z-Average and Polydispersity index (PdI) from DLS measurements are given. The averaged values with standard deviations from three experiments are given (n=3).

| Coupling strategy | Material | Maximum of LSPR band in nm | Δλ in nm | Hydrodynamic diameter in nm | Layer thickness in nm | Z-Average | PdI |
| --- | --- | --- | --- | --- | --- | --- | --- |
| DCP-C | AuNP (20 nm) + Ab in PBS | 536 ± 3 | 14 ± 3 | 85 ± 29 | 32 ± 15 | 204 ± 73 | 0.30 ± 0.05 |
| DCT-C | AuNP (20 nm) + Ab in TRIS | 540.7 ± 1.5 | 19 ± 1.5 | 64 ± 23 | 22 ± 12 | 314 ± 200 | 0.74 ± 0.2 |
| UV-C | AuNP (20 nm) + UV irradiated Ab | 530.7 ± 1.2 | 9 ± 1.2 | 41 ± 7 | 11 ± 4 | 148 ± 59 | 0.47 ± 0.18 |
| TCEP-C | AuNP (20 nm) + TCEP reduced Ab | 533.3 ± 0.6 | 11 ± 0.6 | 48 ± 4 | 14 ± 2 | 109 ± 270 | 0.32 ± 0.06 |
| PEG-C | AuNP (20 nm) + PEG + Ab | 540 ± 7 | 18 ± 7 | 102 ± 24 | 41 ± 12 | 268 | 0.22 ± 0.06 |
| SA-C | AuNP (40 nm) + Streptavidin + biotinylated Ab | 535.3 ± 0.6 | 13 ± 0.6 | 44 ± 5 | 9 ± 3 | 57 ± 4 | 0.296 ± 0.016 |
| DCM-C | AuNP (40 nm) + Ab from microcoat | 539.7 ± 0.6 | - | 72 ± 7 | - | 87 ± 12 | 0.26 ± 0.008 |

**Fig. S3**: Signal at zero amitriptyline concentration for different anti-amitriptyline gold nanoparticle conjugates. The values are calculated as the mean intensity from four channels with their standard deviation as error bars (n=4).

**Fig. S4**: Triplicate calibrations for the different conjugates with logistic calibration curves. The intensities were calculated as normalized signal of test line divided by the signal of the control line. A four-parameter logistic fit was used as calibration function; a) conjugate DCP-C by produced by direct coating in PBS buffer, b) DCT-C produced by direct coating in TRIS buffer, c) TCEP-C produced by direct coating with TCEP reduced antibody, d) PEG-C produced by covalent bond between pegylated AuNP and antibody, e) SA-C produced by binding biotinylated antibody to streptavidin-gold, f) DCM-C produced by Microcoat with the direct coating method.

**Fig. S5**: Stability of the conjugates UV-C and DCM-C on test strip, 250 µl PBS were applied on the sample pad. a) test strip with UV-C, b) decreased signal intensity on test strip with UV‑C after > 12 months of storage, c) test strip with DCM-C, d) decreased signal intensity on test strip with DCM-C after > 9 months of storage.
